# The Global Burden of Suicidal Behavior Among People Experiencing Food Insecurity: A Systematic Review and Meta-analysis

**DOI:** 10.1192/j.eurpsy.2024.302

**Published:** 2024-08-27

**Authors:** M. M. M. Kaggwa

**Affiliations:** Department of Psychiatry and Behavioral Neurosciences, mcmaster University, Hamilton, Canada

## Abstract

**Introduction:**

Food insecurity has become a growing burden within a global context where climate change, catastrophes, wars, and insurgencies are increasingly prevalent. Several studies have reported an association between suicidal behaviors (i.e., suicide ideation, plans, and attempts) and food insecurity. This meta-analytic review for the first time, synthesized the available literature to determine the pooled prevalence of suicidal behaviors among individuals experiencing food insecurity, and examined the strength of their association.

**Objectives:**

To determine the pooled prevalence of suicidal behaviors among individuals experiencing food insecurity, and examine the strength of their association.

**Methods:**

Databases (*Ovid, PubMed, Web of Science, and CINAHL*) were searched using the appropriate search term from inception to July 2022. Eligible studies reporting the number/prevalence of suicidal behaviors among individuals experiencing food insecurity or the association between food insecurity and suicidal behaviors were included. The pooled prevalence of suicidal behaviors was determined using the random-effects model. The review was registered with PROSPERO (CRD42022352858).

**Results:**

A total of 47 studies comprising 75,346 individuals having experienced food insecurity were included. The pooled prevalence was 22.3% for suicide ideation (95% CI: 14.7-29.9; *I* ^2^=99.6%, *p*<0.001, k=18), 18.1% for suicide plans (95% CI: 7.0-29.1; *I* ^2^=99.6%, *p*<0.001, k=4), 17.2% for suicide attempts (95% CI: 9.6-24.8; *I* ^2^=99.9%, *p*<0.001, k=12), and 4.6% for unspecified suicidal behavior (95% CI: 2.8-6.4; *I* ^2^=85.5%, *p*<0.001, k=5). There was a positive relationship between experiencing food insecurity and (i) suicide ideation (aOR=1.049 [95% CI: 1.046-1.052; *I* ^2^=99.6%, *p*<0.001, k=31]), (ii) suicide plans (aOR=1.480 [95% CI: 1.465-1.496; *I* ^2^=99.1%, *p*<0.001, k=5]), and (iii) unspecified suicide behaviors (aOR=1.133 [95% CI: 1.052-1.219; *I* ^2^=53.0%, *p*=0.047, k=6]). However, a negative relationship was observed between experiencing food insecurity and suicide attempts (aOR=0.622 [95% CI: 0.617-0.627; *I* ^2^ = 98.8%, *p*<0.001, k=15]). The continent and the countries income status where the study was conducted were the common cause of heterogeneity of the differences in the odds of the relationships between experiencing food insecurity and suicidal behaviors - with North America and high-income countries (HICs) having higher odds. For suicide attempts, all non HICs had a negative relationship with food insecurity.

**Conclusions:**

There is a high prevalence of suicidal behaviors among individuals experiencing food insecurity. Initiatives to reduce food insecurity would likely be beneficial for mental wellbeing and to mitigate the risk of suicidal behaviors among population experiencing food insecurity.

The paradoxical finding of suicide attempts having a negative relationship with food insecurity warrants further research.

**Disclosure of Interest:**

None Declared

